# Computed Tomography Angiography (CTA) before Reconstructive Jaw Surgery Using Fibula Free Flap: Retrospective Analysis of Vascular Architecture

**DOI:** 10.3390/diagnostics11101865

**Published:** 2021-10-11

**Authors:** Michael Knitschke, Anna Katrin Baumgart, Christina Bäcker, Christian Adelung, Fritz Roller, Daniel Schmermund, Sebastian Böttger, Hans-Peter Howaldt, Sameh Attia

**Affiliations:** 1 Department of Oral and Maxillofacial Surgery, Justus-Liebig-University, Klinikstrasse 33, 35392 Giessen, Germany; Anna.Baumgart@ymail.com (A.K.B.); Christina.Baecker-2@dentist.med.uni-giessen.de (C.B.); Daniel.Schmermund@uniklinikum-giessen.de (D.S.); Sebastian.Boettger@uniklinikum-giessen.de (S.B.); HP.Howaldt@uniklinikum-giessen.de (H.-P.H.); Sameh.Attia@dentist.med.uni-giessen.de (S.A.); 2 Department of Radiology, Justus-Liebig-University, Klinikstrasse 33, 35392 Giessen, Germany; Christian.Adelung@radiol.med.uni-giessen.de (C.A.); fritz.c.roller@radiol.med.uni-giessen.de (F.R.)

**Keywords:** oral cancer, head and neck tumor, fibula free flap, virtual surgical planning

## Abstract

Computed tomography angiography (CTA) is widely used in preoperative evaluation of the lower limbs’ vascular system for virtual surgical planning (VSP) of fibula free flap (FFF) for jaw reconstruction. The present retrospective clinical study analysed *n* = 72 computed tomography angiographies (CTA) of lower limbs for virtual surgical planning (VSP) for jaw reconstruction. The purpose of the investigation was to evaluate the morphology of the fibular bone and its vascular supply in CTA imaging, and further, the amount and distribution of periosteal branches (PB) and septo-cutaneous perforators (SCPs) of the fibular artery. A total of 144 lower limbs was assessed (mean age: 58.5 ± 15.3 years; 28 females, 38.9%; 44 males, 61.1%). The vascular system was categorized as regular (type I-A to II-C) in 140 cases (97.2%) regarding the classification by Kim. Absent anterior tibial artery (type III-A, *n* = 2) and posterior tibial artery (type III-B, *n* = 2) were detected in the left leg. Stenoses were observed mostly in the fibular artery (*n* = 11), once in the anterior tibial artery, and twice in the posterior tibial artery. In total, *n* = 361 periosteal branches (PBs) and *n* = 231 septo-cutaneous perforators (SCPs) were recorded. While a distribution pattern for PBs was separated into two clusters, a more tripartite distribution pattern for SCPs was found. We conclude that conventional CTA for VSP of free fibula flap (FFF) is capable of imaging and distinguishing SCPs and PBs.

## 1. Introduction

Fourteen years after the first description of the free fibula flap (FFF) by Taylor in 1975 [1], the FFF was used for mandibular reconstruction by Hidalgo [2]. This flap is reliable and widely applicable in reconstructive surgery [3]. It offers the possibility of reconstructing both bony and soft tissue defects with a free flap from only one donor site. The FFF can be shaped to almost an ideal form of the missing parts of the jaw and represents the gold standard in mandibular reconstruction [4]. Additionally, it increases the patient’s quality of life after ablative cancer surgery [5,6]. The osseous FFF facilitates prosthetic rehabilitation with dental implants with stable long-term results [7,8].

The vascular supply of the fibula flap is based on the fibular artery (FA), which arises from the truncus tibiofibularis (TTF) after branching the posterior tibial artery (PTA). The truncus continues as the popliteal artery (PA) after debranching the anterior tibial artery (ATA). Preoperative imaging of the vascular status of the lower limb is mandatory for the diagnosis of any anatomical variants. Hypo- or aplasia of the typical three-vessel architecture of the lower leg is crucial to prevent critical limb ischemia [9,10,11]. Peripheral arterial occlusive disease (PAOD) and stenoses have been mentioned as contraindications for flap raising, as they may lead to critical ischemia of the donor-site leg [12,13,14].

Computed tomography angiography (CTA) [15,16,17,18], digital subtraction angiography (DSA) [9,19], or magnetic resonance angiography (MRA) [9,12,17,20,21] are often used as objective techniques for visualization of the lower limbs vascular status. Today CTA and MRA often replace invasive catheter arteriography [14]. Present nephrocytotoxic effects of iodinated contrast media and exposure to radiation are disadvantages of CTA and DSA. Acute renal failure is assigned to endothelial cell damage resulting in endothelial dysfunction [22]. There is a correlation between dosage and an increased risk of renal dysfunction in predisposed patients with impaired renal function (e.g., diabetic patients) [23,24,25]. Reduction of the contrast agent volume would minimize damage to renal function and systemic toxicity [26]. CTA offers numerous advantages as a non-invasive imaging system in comparison to DSA with treating complications of intraarterial application (pseudoaneurysm, arteriovenous fistula) [27]. CTA arises from the standard imaging of the infra-popliteal system in PAOD diagnosis [28]. Further, CTA has been reported to be superior to MRA for visualization of the perforator system [29], and is frequently available, sufficiently precise, and cost-effective [16,30,31]. Other authors prefer MRA as a radiation-free, non-invasive diagnostic, and operator-independent tool [32,33]. In addition, a systematic review, which compared both methods for PAOD diagnosis, found no significant differences [34].

Currently, virtual surgical planning (VSP) and facilitating custom-made, titan-laser-melted patient-specific osteosynthesis plates, CT scans, and DICOM data sets of the donor and recipient site are obligatory [35,36,37]. Performing pre-operative MRA and CTA as standard evaluation before jaw reconstruction will be cost and time expensive. The three-dimensional design and the configured plate allow the translation from virtual planning to surgery site [38]. Due to this planning method, precisely predictable uni- and poly-segmental bony reconstructions are possible [35,39,40]. In literature, 90–95% of FFF success rates were reported in high-volume reconstructive centers [41,42,43,44].

Despite those significant advantages, surgery remains challenging in modifying the planned flap design or a prepared patient specific implant (PSI); if perforator vessels are insufficient, unforeseen difficulties on the vascular bundle occur, or the prepared oncologic resection margins are inadequate [45,46]. The necessity for exploring the contralateral leg and/or dissection of an additional microvascular flap can arise. To minimize this risk, extensive preoperative analysis of the vascular system is necessary, and CTA should be run before every fibula harvest to increase flap safety [47]. CTA facilitates simultaneous evaluation of the bone and vascular system. The preoperative planning for an ideal defect-related skin paddle based on cutaneous perforator vessels is of current research interest. Based on preoperative CTA, assessment of position and run-off of the small vessels has to be performed to design a fibular cutting guide that includes those perforators to increase flap safety [15]. Clinical trials have been carried out on the feasibility of integrating Doppler sonography to detect and locate perforator vessels of the lower leg and facilitate manual transfer to VSP software for presurgical planning [48].

The present retrospective clinical study aimed to determine whether the prospective CTA provides additive aspects for FFF segment molding for VSP. Thus, the purpose of the investigation was to evaluate the morphology of the fibular bone and its vascular supply (lower limb vascular architecture, FA run-off, and distance to the fibula) in CTA imaging, and further, the amount and distribution of PBs and SCPs of the FA. Furthermore, this study aimed to give a clear answer to the following questions:

1. What is the prevalence of vascular anomalies in the present sample?

2. Is it possible to record and distinguish periosteal branches (PBs) and septo-cutaneous perforators (SCPs) of the FA, and up to which diameter can these vessels be detected in routinely run preoperative CTA for VSP?

3. What is the frequency and distribution of PBs and SCPs of the FA?

## 2. Materials and Methods

### 2.1. Study Design and Patient Population

**Figure 1:**
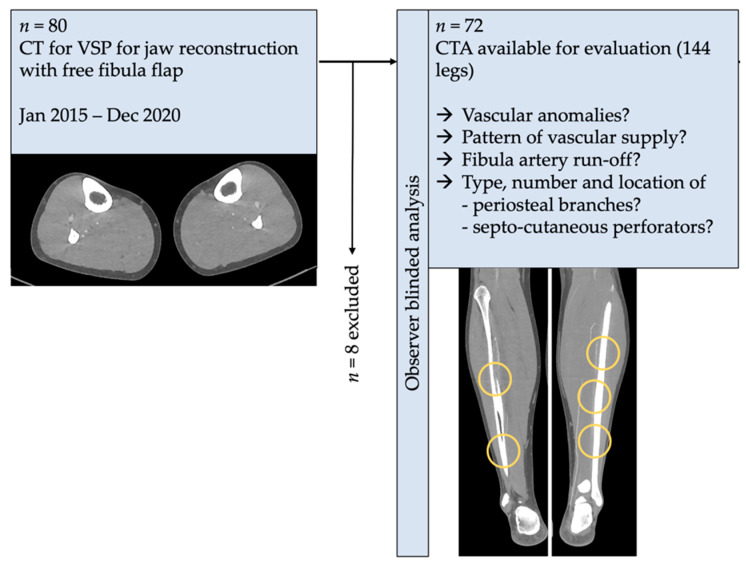
Schematic workflow of the present study. Only computed tomography angiography (CTA) DICOM-datasets of virtual planned jaw reconstructions were included in the investigation. The architecture of lower leg vascular perfusion was analyzed. Number, type of perforator (periosteal branch, septo-cutaneous), and distance to the distal tip of the fibula and between the branches were assessed.

The investigation was conducted as a monocentric, retrospective clinical study on patients who received virtual planned for immediate or delayed jaw reconstruction with FFF from January 2015 to December 2020. The prospective (pre-operative) CTA of the lower limbs was analyzed retrospectively (Figure 1). Out of a total sample size of *n* = 80 VSP cases, 77 CTA datasets of the lower limb were evaluable (MRA instead of CTA, *n* = 3). Of those, five instances of repeated reconstruction after primary flap graft loss (*n* = 4) and tumor recurrence (*n* = 1) were excluded because of one vessel CTA. Finally, a number of (*n* = 72) cases was available for the analysis. The blinded observer was not given any information concerning the elected donor site for FFF transplant.

### 2.2. Inclusion and Exclusion Criteria for Study Subjects

Patients with virtual surgical planning of immediate or delayed jaw reconstruction with FFF and pre-operative CTA and a layer thickness of ≤1.5 mm were enrolled. When previous (VSP) FFF failed and total flap loss occurred, only the datasets of the initial reconstruction attempt with FFF (two fibulae CTA) were included.

### 2.3. Methods, Study Parameters, and Evaluator Calibration

CTA analyses of pseudo-anonymized DICOM data sets (including gender and patient’s age at CTA scan) were performed by a blinded investigator (A.K.B.).

All CTA studies were performed in the Department of Diagnostic and Interventional Radiology of the University Hospital Giessen. The CTA scans were performed on a first- and third-generation dual-energy CT (SOMATOM Definition AS & Force, Siemens Healthineers, Forchheim, Germany). The CTAs used the following standard operating procedure: The first acquisition determined the level for bolus tracking and the scan length. The examination of both legs was done with a slice thickness ≤ 1.5 mm above the aortic bifurcation (chest vertebral body 12) to the feet (70 kV, 300 mA max, pitch 0.5, collimation 0.6 mm, matrix size 512 × 512).

The scans were started when the enhancement peak was observed, and the Bolus tracking approach was utilized to determine the contrast media filling of the relevant vessels. The zone of interest for monitoring vessel filling was positioned in the abdominal aorta at a set level, centered within the artery, and sized to contain only the lumen. Non-ionic contrast medium containing 350 mg of iodine per milliliter (Ultravist 370, Bayer, Leverkusen, Germany) was injected intravenously into an antecubital vein using an 18-gauge needle and a power injector at maximum flow rates of 4.5 or 5.0 mL/s. For a typical scan period of 40 s, the volume of contrast material ranged from 100 to 120 mL (adjusted to patient’s weight).

CTA DICOM data sets were analyzed in HOROS-Software for Macintosh (Version 4.0.0 RC5, Horosproject). Horos is a free and open-source code software (FOSS) program distributed free of charge under the LGPL license at Horosproject.org and sponsored by Nimble Co LLC d/b/a Purview in Annapolis, MD, USA.

The CTA quality was assessed by side-by-side comparison with a region of interest (ROI) in the center of the popliteal artery and dorsal vessels of the dorsum of the foot. For every CTA, the measurements were performed on both patient’s legs.

**Figure 2:**
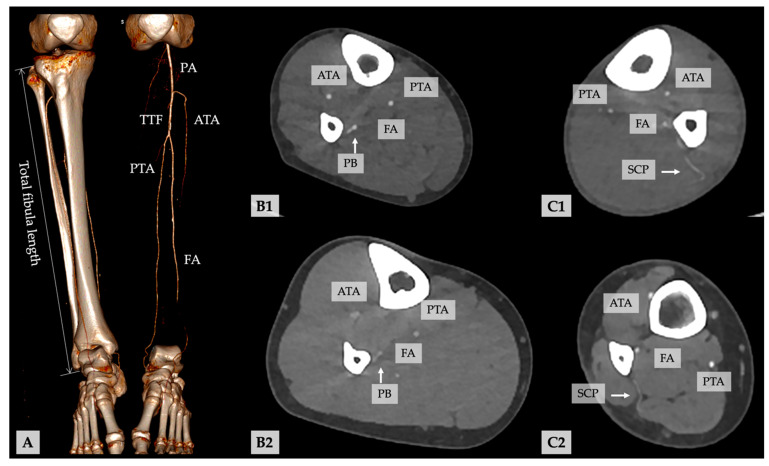
(**A**) Volume rendering and virtual excision of the left tibia and fibula illustrate the vessel run-off along the lower leg. (**B1**,**B2**) Representative examples of periosteal branches (PB) and (**C1**,**C2**) septo-cutaneous perforators (SCP) of the FA in the axial CTA planes (ATA, anterior tibial artery; FA, fibular artery; PA, popliteal artery; PTA, posterior tibial artery; TTF, truncus tibiofibularis).

Based on axial acquired data, the multiplanar three-dimensional reconstructions were performed. Maximum intensity projection (MIP) [49,50] and volume-rendered technique (VRT) reconstructions were used. When MIP and VRT reconstructions are coupled [51], precise identification of tiny vessels in the axial plane, as well as determination of pedicle length and diameters, is achievable. The maximal length of the fibular bone was assessed (Figure 2). The images were studied in the coronal and axial plane for the presence of relevant pathologies (stenoses, PAOD) or anatomic anomalies of the infrapopliteal vascular system. The findings were recorded regarding their localization. The vascular anatomy of the lower limb was categorized concerning the infrapopliteal branching pattern classification described by Kim et al. [52]. The length of the TTF, distance after branching of ATA to the bifurcation of PTA, and FA were measured.

The localization of the main visible (musculo-)septo-cutaneous perforators and periosteal branches (PB) of the FA were identified in the axial plane on CTA (Figure 2), and the distance of the exit of the perforators of the FA to the distal tip of the fibula was recorded. The shortest distance between the surface of the fibular bone and the center of the FA and the diameter of the FA were recorded in 0.5 mm intervals and 50 mm above the distal tip of the fibula bone. The internal diameter of perforators was also recorded. In cases of doubt, impartial, experienced radiologists (C.A. and F.R.) were consulted.

The following parameters were assessed:

Fibula length, bone and vascular anomalies, vascular anatomy and branching pattern of the calf [52], length of the TTF, the shortest distance between the surface of fibular bone and center of the fibular artery (FA) in the axial plane, the internal diameter of the FA, and the number and length of skin perforators and bone feeders from the distal tip of the fibular bone to branching and between the branches (Figure 2). All measurements were recorded in millimeters. Body weight and height were collected from the medical records.

### 2.4. Statistical Analyses

Pearson’s *χ*^2^ test, Fisher’s exact test, and the Freeman-Halton extension [53] were conducted on the categorical variables. The continuous parameters (age, total length of the fibula, length of the FA from origin to the distal tip of the fibula bone, diameter of the FA, length and diameter of the TTF, number and distance of SCPs, and PBs) were verified for normality. The distribution was presented as a mean (standard deviation), and Student’s *t*-test was performed. *p* < 0.05 was defined as statistically significant. The statistical analysis was carried out with SPSS 25 (SPSS Inc., Chicago, IL, USA).

### 2.5. Ethics Statement/Confirmation of Patients’ Permission

The local Ethics Committee of Justus-Liebig-University Giessen approved the study (AZ33/20), and patients’ permission/consent was not necessary for this retrospective study. All collected data in the Microsoft Excel spreadsheet were pseudonymized.

## 3. Results

A total of 72 patients (28 females, 38.9%; 44 males, 61.1%) fulfilled the inclusion criteria. The mean age was 58.5 ± 15.3 years (range: 14.8–82.6 years). A series of eight virtual planned reconstructions were excluded due to missing data (MRA instead of CTA, *n* = 3, one leg CTA: reconstruction with contralateral fibula after flap loss, *n* = 4 and after tumor recurrence, *n* = 1).

A total of 144 lower limbs were assessed. The groups were tested for normal distribution. More than half (61.1 %, *n* = 44), of the included study participants were male. Their mean age was 56.8 ± 14.6 years, and they were 4.3 years younger than the female patients; the difference was non-significant (*p* = 0.257). The length of the fibula revealed a gender-specific, significant difference within the mean near to 45 mm more prominent bone in males (*p* ≤ 0.001). The detailed demographic parameters and results are summarized in Table 1.

**Figure 3:**
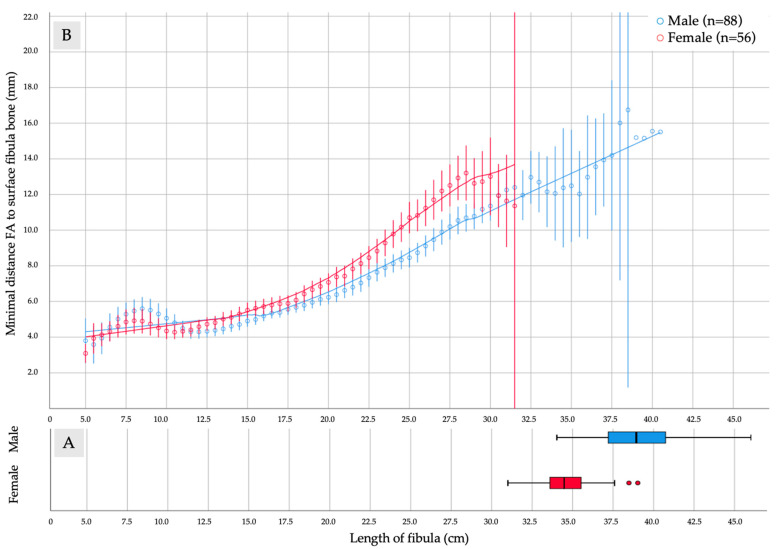
The diagram summarizes assessed parameters of (**A**) length of fibula and (**B**) distance and length of the fibular artery (FA) in relation to fibular bone surface in CTA of 144 lower limbs in 72 patients. (**A**) A significant difference for total length of the fibular bone was measured (male: *n* = 88, mean ± SD: 39.08 ± 2.35 cm vs. female: *n* = 56, mean ± SD: 34.60 ± 1.70 cm; *p* = 0.0001). (**B**) Comparison of the minimal distance of FA run-off (mm) in males (*n* = 88) and females (*n* = 56) measured in axial planes from the distal tip of the fibula (cm). LOESS curves with the inclusion of 50% of the assessed points were drawn.

The run-off of the fibular artery (FA) concerning the surface of the fibular bone and the total length is depicted gender-separated in Figure 3A,B. A significant difference in total length of the fibular bone was found (male: *n* = 88, mean ± SD: 39.08 ± 2.35 cm vs. female: *n* = 56, mean ± SD: 34.60 ± 1.70 cm; *p* ≤ 0.001). Despite the observed difference of the gender- and body-height-dependent run-off of the FA, the LOESS curves with the inclusion of 50% of the assessed points run almost congruent in the interval between 5.0 cm and 15.0 cm. While between 5.0 cm and 11.0 cm, the FA was found nearer to the surface of the fibula in females than males, by over 11.0 cm to 15.0 cm, the FA was detected, on average, 1.0 mm in the median and the proximal third and more than 2.0 mm farther distant to the fibula in females than in males (Figure 3B).

The gender characteristic difference was also found for the length of FA (*p* ≤ 0.001), and diameter of the FA (male: *n* = 88, mean ± SD: 3.41 ± 0.78 mm vs. female: n = 56, mean ± SD: 2.78 ± 0.64 mm; *p* ≤ 0.001). Analogous gender-associated results were found for the length (male: *n* = 80, mean ± SD: 35.15 ± 14.07 mm vs. female: *n* = 48, mean ± SD: 28.20 ± 11.51 mm; *p* = 0.045) and diameter (male: *n* = 80, mean ± SD: 4.44 ± 1.00 mm vs. female: *n* = 48, mean ± SD: 3.64 ± 0.65 mm; *p* ≤ 0.001) of TTF in the type I-A branching pattern of the infra-popliteal vessels (Table 1).

**Figure 4:**
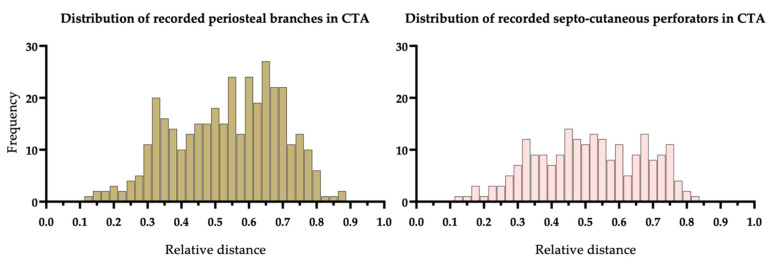
(**Left**) Distribution of periosteal branches (*n* = 361) and (**Right**) septo-cutaneous perforators (*n* = 213) of the FA demonstrated by the CTA. The origin of the vessels is scaled concerning the total length of the fibula and given as relative distance.

A total number (*n* = 361) of periosteal branches (PB) and (*n* = 231) septo-cutaneous perforators (SCP) was recorded. The distribution of CTA-based detected PB and SCP is shown in Figure 4. The histogram of scaled vessel origins demonstrates a bi- for PB and trimodal distribution for SCP. The distal PB cluster was narrow, with its maximum placed around 0.30 RD (relative distance), and the proximal one is broader with a frequency peak between 0.55 RD and 0.70 RD. A tripartite distribution pattern was found in SCPs. The distal clustering was narrow and located around 0.30 RD. The mean clustering was broad and located between 0.45 RD and 0.60 RD. Proximally, a third cluster appeared at 0.70 RD. Insignificant differences were recorded concerning gender and diameter of PB (*p* = 0.472) and SCP (*p* = 0.233) of the FA. The diameter of PB ranged from 0.35 mm to 2.26 mm (male: *n* = 230, mean ± SD: 0.88 ± 0.25 mm vs. female: *n* = 131, mean ± SD: 0.86 ± 0.26 mm; *p* = 0.472). SCP ranged from 0.52 mm to 2.43 mm (male: *n* = 127, mean ± SD: 0.91 ± 0.26 mm vs. female: *n* = 86, mean ± SD: 0.96 ± 0.35 mm; *p* = 0.233). The diameter of both vessel types was measured at the origin of the FA (Table 1).

The evaluated parameters of the lower leg and the vascular system are summarized in Table 2. In a side-by-side comparison, non-significant differences for the total length of the fibular bone were assessed (*p* = 0.953). One case of a healed isolated fibula fracture was found. In the investigated sample of *n* = 144 lower legs, the vascular system was categorized as regular (type I-A to II-C) in 140 cases (97.2%) [52]. Absent ATA (type III-A, *n* = 2) and PTA (type III-B, *n* = 2) were detected in the left leg (Table 3). Stenoses were mostly observed in FA (*n* = 11), once in the ATA, and twice in the PTA. While the number of detected SCP of the FA were nearly similar (left: *n* = 103; right: *n* = 108), a remarkable difference of 12.5% was observed concerning the number of PB on the right leg (left: *n* = 168; right: *n* = 193). The average diameter of PB differed significantly in side-to-side comparisons (*p* = 0.033) and was larger in the right lower leg.

The study found an average amount (±SD) of 2.51 ± 1.55 (median: 3.0; range: 0–7) PB and 1.48 ± 1.12 (median: 1.0; range: 0–4) SCP with a mean (±SD) distance between them of 5.17 ± 3.07 cm (median: 4.6 cm; range: 0.1–15.5 cm) of the FA in each fibula in the region of interest between the origin of FA from TTF and 5.0 cm above the distal tip of the fibular bone. One PB was found in 10.77% of the 1 cm segments, 21.07% of the 2.0 cm segments, and 29.17% of the 3 cm segments. One SCP was found in 6.46% of the 1 cm segments, 12.25% of the 2.0 cm segments, and 17.84% of the 3 cm segments (Table 4).

## 4. Discussion

Over the past three decades, there has been debate about the best technique for vascular evaluation of fibular grafts [54], which was abandoned in favor of the CT for VSP, which can easily be modified as CTA. Numerous investigations have shown the impact of CTA as a sensitive and specific method for microsurgical free flap [15,16,17,18] and perforator flap harvesting in reconstructive surgery [55,56,57,58,59,60,61,62,63].

### 4.1. What Is the Prevalence of Vascular Anomalies in the Present Sample?

In the evaluated sample of *n* = 144 lower limbs, a majority of 88.9% (*n* = 128) were assigned as type I-A concerning the classification by Kim [52], and four limbs (2.8%) with dominant FA variants (type III-A: *n* = 2 and III-B: *n* = 2) were found. The results are comparable to the literature [64]. The sample revealed no cases of a peronea arteria magna (type III-C), in which FA is the main blood-supplying vessel for the lower limb and foot.

Before FFF harvesting, it is crucial to detect this specific, singular vasculature to prevent significant and critical limb and foot ischemia [13,65,66]. In comparison to the vascular supply of the forearm by the superficial and deep palmar arterial arch, for which sufficient perfusion is evaluable by Allen’s test, anatomical anomalies like type III-A-C elude simple clinical examination [12,67,68,69]. In type III-A and -B, blood supply of the foot is shared between the FA and non-hypoplastic ATAs or PTAs, and the diameter of the FA is then often enlarged [14,68,70]. The prevalence of such a dominant FA was published with 5.2% of any leg, and furthermore, critical vascular anomalies of the lower limb were recorded in 10% of the population [17]. The findings of the present study show dominant FA variants in 2.8% of the investigated sample. Vascular anomalies (stenoses *n* = 14) had been recorded in 9.7% in the present investigation and were well comparable to the study [17]. Stenoses were mainly found in *n* = 11 FAs.

Other studies found that the FA is less severely affected by the PAOD than the tibial arteries [11,12,54]. For its diagnosis, the ankle-brachial index (ABI) is reported as a cheap and non-invasive test [71]. Suspicious scores of 0.9 or less implicate further diagnosis of PAOD [72,73,74] by MRA or CTA [75,76]. According to the literature, findings of preoperative clinical examination of the vascular system with ABI were not predictive of a problem when color flow Doppler sonography and angiography results were not physiological [77,78,79]. A combined ABI and handheld Doppler sonography examination were not accurate enough and are not sufficient for developing the surgical plan for a FFF [78].

Further investigations found that pathologic ABI was related to difficulty with the microvascular anastomosis [80]. Furthermore, the wide variability concerning sensitivity and specificity of the ABI test must be considered [81]. Because of insufficient evidence, US Institutions do not recommend the ABI test for PAOD screening in asymptomatic individuals [82]. However, in the present study the ABI test was not performed in our preoperative routine.

### 4.2. Is It Possible to Record and to Distinguish Periosteal Branches and Septo-Cutaneous Perforators of the FA, and up to Which Diameter Can These Vessels Be Detected in Routinely Run Preoperative CTA for VSP?

The evaluated prospective—in daily routine—CTA studies in the present investigation were sufficient to evaluate the lower limb’s vascular system and perforator system. PBs and SCPs were distinguishable by their course and direction to the skin. Numerous investigations have shown the impact of CTA as a sensitive and specific method for microsurgical free flap [15,16,17,18,83] and perforator flap harvesting in plastic and reconstructive surgery [55,56,57,58,59,60,61,62,63]. Previous studies on lower limbs found that CTA demonstrated all the size, course, and penetration patterns of perforators over 0.3 mm in diameter [16]; these patterns were demonstrated more clearly in perforators with a diameter larger than 1 mm [56,62]. In the present study, the mean diameter of SCPs was 0.93 ± 0.30 mm (range: 0.52–2.43 mm) and of PBs, 0.87 ± 0.25 mm (range: 0.35–2.26 mm). Other studies found that CTA is accurate for estimating fibular length, run-off, and course of the infra-popliteal vasculature and the perforator subsystem but less precise in predicting perforator vessel diameter [31]. The disadvantage is, in our experience without clinical contribution because of the dissection of the posterior intermuscular septum on-sight and inclusion of a small cuff of soleus muscle for a safe and reliable supply of the skin paddle (musculo-septo-cutaneous perforators).

Therefore, it is crucial, that the CTA method in our setting can mark/visualize small vessels down to a diameter 0.35 mm in routine CTA for VSP. The rate of undetected PBs and SCPs remains unclear. Preoperative information about the location and course of those vessels could be helpful for planning and achieving reliable single or bi-partitioned skin paddles [32,84] and poly-segmental jaw reconstructions [83].

### 4.3. What Is the Frequency and Distribution of PBs and SCPs of the FA?

The results of the study show different distribution patterns for PBs and SCPs. A more extended bone section increases the probability of finding a perforator. PBs were encountered with about twice the frequency of SCP. Of particular note is the observation that for a fibula segment of 3.0 cm in length, a PB was found on average 29.17% and a SCP in 17.84% in CTA assessment. However, the observed distribution patterns reflect PB and SCP clustering and confirm the high variability of the localization and course. We found a bimodal distribution pattern for PBs and three peaks for SCPs in performed CTA for VSP. These patterns are comparable to the results of other studies [21,31,85].

Published literature shows that CT scans after barium latex mixture in fresh frozen cadaver lower limbs showed in mean 12.8 periosteal branches of the fibula artery with a mean distance of 1.36 cm between them [86]. It was observed that one branch was found in 65.1% in 1.0 cm segments, in 83.4% of 1.5 cm segments, and 94% of the 2.0 cm segments. Another CTA in vivo study demonstrated all perforator size, course, and penetration patterns were over 0.3 mm in diameter [16]. Present study findings show different results. Only in 10.77%, was one PB found in a 1.0 cm fibula segment in our defined region of interest between the origin of the FA and a plane 5 cm above the distal tip of the fibula. The likelihood increased in 2.0 cm segments to 21.07% and in 3.0 cm segment lengths to 29.17%, having included at least one PB (Table 4). However, a comparison of the results of the cadaver study is only limited, possibly because of the differences in method, radiation dosage, and type and application of the contrast agent [86]. Nevertheless, the findings support the possibility of including even small segments in jaw reconstruction. For VSP our team avoids segment length less than 30 mm to achieve sufficient bone segment perfusion to prevent partial and total flap loss.

Previous studies found that the skin paddle of the FFF can be categorized into four subtypes concerning its vascular supply. Detailed information about the vascular pattern of the skin paddle is required, especially to salvage larger paddles [87]. The course of the perforator system is crucial in difficult situations (e.g., loss or discharge of previous graft) for precise planning. CTA imaging allows individual mapping of the vasculature.

### 4.4. Limitations of the Study

Our study sample was formed by benign and malign pathologies and included immediate and delayed jaw reconstructions with VSP FFF. The performed CTAs had not been run under experimental conditions but in clinical routine. Intraoperative matching would be of great value to validate the number and distribution of PBs and SCPs observed using CTA, but the study cannot give an answer about the number of undetected PBs and SCPs in our sample. It has to be expected that several PBs and SCPs were not observed in the CTA evaluation. Further clinical investigations are necessary for this purpose.

### 4.5. Implications

While the present study evaluated the vascular architecture of the lower limb independent of flap outcome, further investigations on the study sample are necessary to assess the following questions:

1. How do fibular artery stenoses effects the outcome of flap surgery?

2. How does the distribution of CTA- detected PBs and SCPs influence the surgical result of mono- and poly-segmental jaw reconstructions, as well as partial and total flap loss?

3. Does the observed distribution of PBs and SCPs impact wound healing of the donor site?

It is planned to match the study results with the virtual surgical planning, and further to analyze them regarding flap success and failure.

## 5. Conclusions

Routinely run CTA for virtual surgical planning of free fibula flap (FFF) is capable of imaging and distinguishing septo-cutaneous perforators (SCPs) and periosteal branches (PBs) of the fibula artery. The density and distribution obtained differ from those of anatomical cadaver studies. The more proximal the FFF segment, the more frequently a potential PB was observed in the CTA. Knowledge of perforator location may help to plan segment lengths and outline of skin paddles to avoid impending complications.

## Figures and Tables

**Table 1:** CTA assessment for bone and vascular system parameters of the study group categorized for gender (cm, centimeter; FA, fibular artery; PB, periosteal branch; SCP, septo-cutaneous perforator; SD, standard deviation; TTF, truncus tibiofibularis). Annotation: ^‡^ Length and diameter of TTF were only assessed for type I-A branching pattern.

*n* = 72 Patients	*n* (%)	*n* = 144 Lower Limbs	*p*-Value
Age at CT-scan (years), mean ± SD			
Male	44 (61.1)	56.8 ± 14.6	
Female	28 (38.9)	61.1 ± 16.1	0.257
Body weight (kilogram), mean ± SD			
Male (range)	44 (61.1)	77.7 ± 15.7 (37.5–113.0)	
Female (range)	28 (38.9)	66.5 ± 14.1 (43.2–104.0)	0.003
Body height (cm), mean ± SD			
Male (range)	44 (61.1)	177.5 ± 7.2 (159.8–198.0)	
Female (range)	28 (38.9)	161.4 ± 5.6 (150.0–176.0)	≤0.001
BMI (kg/m^2^), mean ± SD			
Male (range)	44 (61.1)	24.6 ± 4.9 (14.7–40.0)	
Female (range)	28 (38.9)	25.4 ± 5.4 (17.3–40.0)	0.518
Length of fibula, mean (mm) ± SD			
Male (range)	44 (61.1)	390.8 ± 23.5 (340.2–460.1)	
Female (range)	28 (38.9)	346.0 ± 17.0 (310.0–390.2)	≤0.001
Length of TTF, mean (mm) ± SD ^‡^			
Male (range)	80 (62.5)	35.15 ± 14.07 (6.0–67.2)	
Female (range)	48 (37.5)	28.20 ± 11.51 (10.0–64.5)	0.0045
Diameter of TTF, mean (mm) ± SD ^‡^			
Male (range)	80 (62.5)	4.44 ± 1.00 (2.67–6.78)	
Female (range)	48 (37.5)	3.64 ± 0.65 (2.30–5.12)	≤0.001
Length of FA, mean (mm) ± SD			
Male (range)	44 (61.1)	25.66 ± 3.72 (7.0–35.85)	
Female (range)	28 (38.9)	22.42 ± 3.65 (4.65–26.6)	≤0.001
Diameter of FA, mean (mm) ± SD			
Male (range)	44 (61.1)	3.41 ± 0.78 (1.88–5.40)	
Female (range)	28 (38.9)	2.78 ± 0.64 (1.46–4.27)	≤0.001
Diameter of SCP, mean (mm) ± SD			
Male (range)	127 (59.6)	0.91 ± 0.26 (0.53–1.82)	
Female (range)	86 (40.4)	0.96 ± 0.35 (0.52–2.43)	0.233
Diameter of PB, mean (mm) ± SD			
Male (range)	230 (63.7)	0.88 ± 0.25 (0.40–1.89)	
Female (range)	131 (36.3)	0.86 ± 0.26 (0.35–2.26)	0.472

**Table 2:** CTA assessment for the study sample’s fibula bone and vascular system parameters (SD, standard deviation; ATA, anterior tibial artery; FA, fibular artery; PB, periosteal branch; PTA, posterior tibial artery; SCP, septo-cutaneous perforator; TTF, truncus tibiofibularis). Annotation: ^‡^ Length and diameter of TTF were only assessed for type I-A branching pattern.

*n* = 144	Left Fibula *n* = 72	Right Fibula *n* = 72	*p*-Value	Total
Fibula length, mean (mm) ± SD	373.5 ± 30.8	373.2 ± 30.4	0.953	144
Fibula bone anomalies, *n*			-	
Fracture	1	0		1
Infrapopliteal branching pattern [52]			-	
Regular (I-A to II-C)	68	72		140
Absent ATA (III-A)	2	-		2
Absent PTA (III-B)	2	-		2
Stenoses, *n*			-	
ATA	1	-		1
PTA	2	-		2
FA	5	6		11
Length of TTF, mean (mm) ± SD ^‡^	30.37 ± 12.78	35.03 ± 14.26	0.053	128
Diameter of TTF, mean (mm) ± SD ^‡^	4.06 ± 0.92	4.14 ± 0.97	0.663	128
Length of FA, mean (mm) ± SD	244.5 ± 41.3	243.4 ± 39.2	0.809	144
Diameter of FA, mean (mm) ± SD	3.17 ± 0.80	3.16 ± 0.78	1.0	144
Distance to the fibular bone, mean (mm) ± SD	8.58 ± 3.74	8.55 ± 3.81	0.974	144
Overall found SCP, *n*	104	109		213
Mean SCP per fibula (mm) ± SD	1.44 ± 1.10	1.51 ± 1.13	0.647	1.48 ± 1.12
Overall found PB, *n*	168	193		361
Mean PB per fibula (mm) ± SD	2.33 ± 1.46	2.68 ± 1.62	0.033	2.51 ± 1.55

**Table 3:** Infra-popliteal arterial branching variations of the study sample (*n* = 144) were categorized according the classification by Kim et al. [52].

Type *n* (%)	Left Leg, *n* = 72	Right Leg, *n* = 72	All, *n* = 144
I-A	63 (87.5)	65 (90.3)	128 (88.9)
I-B	2 (2.8)	1 (1.4)	3 (2.1)
I-C	1 (1.4)	-	1 (0.7)
II-A	1 (1.4)	2 (2.8)	3 (2.1)
II-B	1 (1.4)	4 (5.6)	5 (3.6)
II-C	-	-	-
III-A	2 (2.8)	-	2 (1.4)
III-B	2 (2.8)	-	2 (1.4)
III-C	-	-	-

**Table 4:** Number (%) of fibular segments that were supplied by periosteal branches (PB, *n* = 361) and corresponding septo-cutaneous perforators (SCP, *n* = 213) of FA of all lower legs (*n* = 144).

Size of Segment (cm)/Vessel Type	None	One	Two or More
1.0 PB	89.05%	10.77%	0.18%
2.0 PB	78.33%	21.07%	0.68%
3.0 PB	68.28%	29.17%	2.55%
1.0 SCP	93.54%	6.46%	0
2.0 SCP	87.22%	12.25%	0.54%
3.0 SCP	81.28%	17.84%	0.88%

## Data Availability

The datasets generated and/or analyzed during the current study are available from the corresponding author upon reasonable request.
